# Supplementary material for: Emotional learning retroactively promotes memory integration through rapid neural reactivation and reorganization
Source: eLife. 2022 Dec 8;11:e60190. doi: 10.7554/eLife.60190 (PMC9815824; doi:10.7554/eLife.60190)
Supplement: Supplementary file 1. [file elife-60190-supp1.docx]

#### **Table S1. Acoustic characteristics of the four voice clips**

|  | Duration (sec) | Frequency (Hz) | | | | Power (db) | | | |
| --- | --- | --- | --- | --- | --- | --- | --- | --- | --- |
|  |  | median | mean ± s.d. | min | max | median | mean ± s.d. | min | max |
| Scream1 | 2 | 419.19 | 350.12 ± 131.29 | 99.79 | 477.44 | 78.15 | 77.64 ± 2.75 | 72.91 | 83.52 |
| Scream2 | 2 | 307.42 | 338.93 ± 82.83 | 198.13 | 514.32 | 78.14 | 74.79 ± 6.51 | 60.07 | 80.76 |
| “Eh” | 2 | 205.76 | 205.75 ± 1.42 | 202.92 | 208.14 | 78.15 | 77.40 ± 1.66 | 72.69 | 79.53 |
| “Ah” | 2 | 214.52 | 212.14 ± 13.76 | 92.23 | 216.96 | 78.13 | 76.73 ± 9.15 | -5.39 | 79.53 |

Notes: Sec, second; Hz, Hertz; db, decibel.
