# Supplementary material for: Emotional learning retroactively promotes memory integration through rapid neural reactivation and reorganization
Source: eLife. 2022 Dec 8;11:e60190. doi: 10.7554/eLife.60190 (PMC9815824; doi:10.7554/eLife.60190)
Supplement: Supplementary file 2. [file elife-60190-supp2.docx]

#### **Table S2. Brain regions involved in emotional effect on condition-level reactivation**

| **Brain Regions** | **Hemisphere** | ***T* values** | **MNI Coordinates** | | |
| --- | --- | --- | --- | --- | --- |
|  |  |  | **X** | **Y** | **Z** |
| **Aversive vs. Neutral** | | | | | |
| Hippocampus | R | 4.88 | 16 | -36 | 8 |
| Parahippocampal gyrus | R | 5.29 | 22 | 6 | -20 |
| Fusiform gyrus | L | 3.90 | -36 | -48 | -16 |
| Insula | R | 4.70 | 36 | -10 | 16 |
| Inferior orbit frontal cortex | L | 6.50 | -44 | 20 | -12 |
| Middle frontal cortex | L | 4.37 | -26 | 44 | 18 |
|  | R | 6.35 | 46 | 0 | 58 |
| Superior medial frontal cortex | R | 6.43 | 12 | 28 | 60 |
| Superior temporal pole | R | 5.41 | 48 | 20 | -22 |
| Middle temporal cortex | R | 5.03 | 40 | -60 | 18 |
|  | R | 5.79 | 64 | -2 | -16 |
| Superior temporal cortex | R | 5.34 | 46 | -34 | 8 |
| Inferior temporal cortex | R | 3.96 | 62 | -52 | -18 |
| Middle occipital cortex | L | 4.55 | -50 | -78 | 8 |
| Precuneus | L | 6.35 | -10 | -60 | 14 |
| Cuneus | R | 5.69 | 16 | -74 | 20 |
| Angular gyrus | R | 5.66 | 32 | -62 | 46 |

Notes: Regions were derived from a whole-brain exploratory analysis using searchlight algorithm on condition-level pattern similarity. Significant clusters, at a height threshold of *p* < 0.005 and an extent threshold of *p* < 0.05 with family-wise error correction for multiple comparisons based on nonstationary suprathreshold cluster-size distributions computed by Monte Carlo simulations, are reported with local maximum *T* statistic in Montreal Neurological Institute (MNI) space. L, left; R, right.
