# Supplementary material for: Emotional learning retroactively promotes memory integration through rapid neural reactivation and reorganization
Source: eLife. 2022 Dec 8;11:e60190. doi: 10.7554/eLife.60190 (PMC9815824; doi:10.7554/eLife.60190)
Supplement: Supplementary file 3. [file elife-60190-supp3.docx]

#### **Table S3. Brain regions involved in emotional effect on hippocampal connectivity during emotional learning**

| **Brain Regions** | **Hemisphere** | ***T* values** | **MNI Coordinates** | | |
| --- | --- | --- | --- | --- | --- |
|  |  |  | **X** | **Y** | **Z** |
| **Aversive vs. Neutral** | | | | | |
| Amygdala | R | 6.24 | 30 | 0 | -22 |
| Fusiform gyrus | L | 4.34 | -28 | -44 | -10 |
| Middle temporal cortex | L | 4.03 | -46 | -70 | 14 |
| Superior temporal cortex | L | 4.12 | -64 | -18 | 12 |
| Medial orbit frontal cortex | R | 4.64 | 8 | 60 | -12 |
| Middle frontal cortex | L | 4.31 | -28 | 40 | 32 |
|  | R | 4.06 | 42 | 54 | 2 |
| Inferior frontal cortex | R | 4.22 | 42 | 16 | 14 |
| Middle cingulate cortex | L | 3.79 | -6 | -24 | 40 |
| Supramarginal gyrus | R | 4.20 | 46 | -38 | 30 |
| Calcarine | L | 3.95 | -24 | -72 | 8 |

Notes: Regions were derived from a PPI analysis on hippocampal functional connectivity during the emotional learning phase. Significant clusters, at a height threshold of *p* < 0.005 and an extent threshold of *p* < 0.05 with family-wise error correction for multiple comparisons based on nonstationary suprathreshold cluster-size distributions computed by Monte Carlo simulations, are reported with local maximum *T* statistic in Montreal Neurological Institute (MNI) space. L, left; R, right.
