# Supplementary material for: Emotional learning retroactively promotes memory integration through rapid neural reactivation and reorganization
Source: eLife. 2022 Dec 8;11:e60190. doi: 10.7554/eLife.60190 (PMC9815824; doi:10.7554/eLife.60190)
Supplement: Supplementary file 4. [file elife-60190-supp4.docx]

#### **Table S4. Post-encoding hippocampal connectivity changes in positive relation to memory in the aversive condition but negative in the neutral condition**

| **Brain Regions** | **Hemisphere** | ***T* values** | **MNI Coordinates** | | |
| --- | --- | --- | --- | --- | --- |
|  |  |  | **X** | **Y** | **Z** |
| **Rest 2 vs. Rest 1** | | | | | |
| Lateral occipital cortex | L | 3.88 | -42 | -66 | -6 |
| Lingual gyrus | R | 5.02 | 10 | -88 | -14 |
| **Rest 3 vs. Rest 2** | | | | | |
| Inferior parietal Lobule | L | 4.16 | -56 | -42 | 40 |
|  | R | 4.09 | 52 | -54 | 40 |
| Superior frontal cortex | R | 4.26 | 24 | 52 | 14 |
| Posterior cingulate cortex | R | 4.22 | 10 | -36 | 40 |
| Medial frontal cortex | R | 4.31 | 10 | 50 | 16 |
| Putamen | L | 4.73 | -20 | 14 | 4 |

Notes: Regions were derived from the multiple regression analyses on post-encoding hippocampal functional connectivity with memory accuracies in aversive and neutral conditions as two separate covariates of interest. Significant clusters, at a height threshold of *p* < 0.005 and an extent threshold of *p* < 0.05 with family-wise error correction for multiple comparisons based on nonstationary suprathreshold cluster-size distributions computed by Monte Carlo simulations, are reported with local maximum *T* statistic in Montreal Neurological Institute (MNI) space. L, left; R, right.
